# An Unusual Case of Renal-Limited Sarcoidosis Presenting With Hypercalcemia of Unknown Origin: A Case Report

**DOI:** 10.7759/cureus.90278

**Published:** 2025-08-17

**Authors:** Pooja Patel, Anjali Satoskar, Armen Margaryan

**Affiliations:** 1 Internal Medicine, Florida Atlantic University Charles E. Schmidt College of Medicine, Boca Raton, USA; 2 Pathology, The Ohio State University College of Medicine, Columbus, USA

**Keywords:** acute kidney injury in elderly, acute renal failure and hemodialysis, extrapulmonary manifestation of sarcoidosis, renal sarcoidosis, severe hypercalcemia, treatment-resistant hypercalcemia

## Abstract

Sarcoidosis is a relatively uncommon multisystem disease that is often difficult to diagnose, frequently requiring multiple physician evaluations. While common symptoms include fatigue, weight loss, lymphadenopathy, and multi-organ involvement, some cases deviate from this classic presentation. We present the case of an elderly male patient who exhibited severe hypercalcemia without the typical systemic manifestations of sarcoidosis. His hypercalcemia of unknown origin led to multiple hospitalizations. Initial medical management provided only temporary relief, and an extensive workup failed to identify a cause. As his condition progressed to acute kidney injury requiring hemodialysis, a more invasive evaluation was pursued. A bone marrow biopsy was unremarkable, but a subsequent kidney biopsy revealed renal-limited sarcoidosis, a rare form of the disease. The patient was started on high-dose corticosteroids, which led to clinical improvement; however, his renal function did not recover sufficiently to discontinue hemodialysis.

## Introduction

Sarcoidosis is a systemic inflammatory disease of unclear etiology that was first described in 1889 by Besnier et al. [1]. It presents as a multisystem granulomatous disorder with accumulation of non-necrotizing granulomas. Studies suggest that patients with sarcoidosis may have a predisposition to the disease due to a chronically activated inflammatory state, which can be present years before clinical symptoms arise [2]. Disease progression involves phagocytosis and presentation of an unidentified antigen by antigen-presenting cells, triggering an exaggerated immune response [3] with granuloma formation. Although no single causative antigen has been identified [4], several potential triggers have been proposed, including infectious organisms, environmental exposures, and occupational agents. The putative antigen is presented to CD4+ T helper cells, which initiate and amplify the immune response, ultimately resulting in granuloma formation [5]. As a result of the exaggerated immune response, patients may experience a wide range of symptoms, including fatigue, weight loss, fever, cough, dyspnea, lymphadenopathy, pulmonary nodules, joint pain, skin lesions, uveitis, and involvement of the liver, spleen, and kidneys [6,7].

Pulmonary involvement is the hallmark of sarcoidosis, affecting up to 90% of patients. Other frequently involved systems include the skin, eyes, liver, and peripheral lymph nodes [8]. Renal involvement occurs in approximately 25%-30% of cases, either as part of systemic disease or as an isolated manifestation [6,9]. Among renal manifestations, granulomatous interstitial nephritis (GIN) is most common, while hypercalcemia-related complications occur in fewer than 10% of cases [6,7]. Rarely, renal sarcoidosis progresses to end-stage renal disease (ESRD) requiring dialysis [9].

Sarcoidosis can affect individuals across racial and gender groups, although it is more prevalent in women and African Americans compared to men and Caucasians [10]. Despite its recognizable features, sarcoidosis remains a diagnosis of exclusion, with more than half of symptomatic patients requiring evaluation by three or more physicians before a diagnosis is confirmed [8]. Renal sarcoidosis can be difficult to diagnose and can go undetected for many years, especially if classic sarcoidosis findings, such as pulmonary involvement, are not present [6].

In this report, we present a case of renal-limited sarcoidosis, initially manifesting as hypercalcemia of unknown origin. The diagnostic process was prolonged due to the absence of classic systemic sarcoidosis findings, ultimately requiring multiple evaluations over six months before the diagnosis was established.

## Case presentation

A 79-year-old White man was admitted on multiple occasions with recurrent hypercalcemia and acute kidney injury. The patient's past medical history is significant for chronic kidney disease (CKD) stage 3b, remote history of transitional cell bladder cancer with metastasis to the right ureter and kidney status-post right nephrectomy, and basal cell carcinoma status-post resection. Initially, the patient presented to his outpatient nephrologist's office for hypercalcemia of 12.4 mg/dL (normal range: 8.6-10.2 mg/dL), with normal serum albumin of 3.8 g/dL (normal range: 3.8-4.8 g/dL) and worsening creatinine of 2.51 mg/dL (baseline: 1.9 mg/dL), which prompted investigation for an etiology. Serum protein electrophoresis and immunofixation, as well as urine immunofixation, were negative for a monoclonal spike. Intact parathyroid hormone (PTH) was noted to be appropriately suppressed at 5 pg/mL (normal range: 16-77 pg/mL), and 25-hydroxyvitamin D was 49.3 ng/mL (normal range: 30-100 ng/mL). However, worsening creatinine of 2.6 mg/dL and hypercalcemia of 13 mg/dL led to hospitalization for urgent treatment. During the hospitalization, he denied fatigue, weight loss, or infectious symptoms, and was hemodynamically stable with an unremarkable physical examination. Intravenous fluids, pamidronate, and calcitonin led to the resolution of hypercalcemia, and the patient was discharged home.

**Figure 1:**
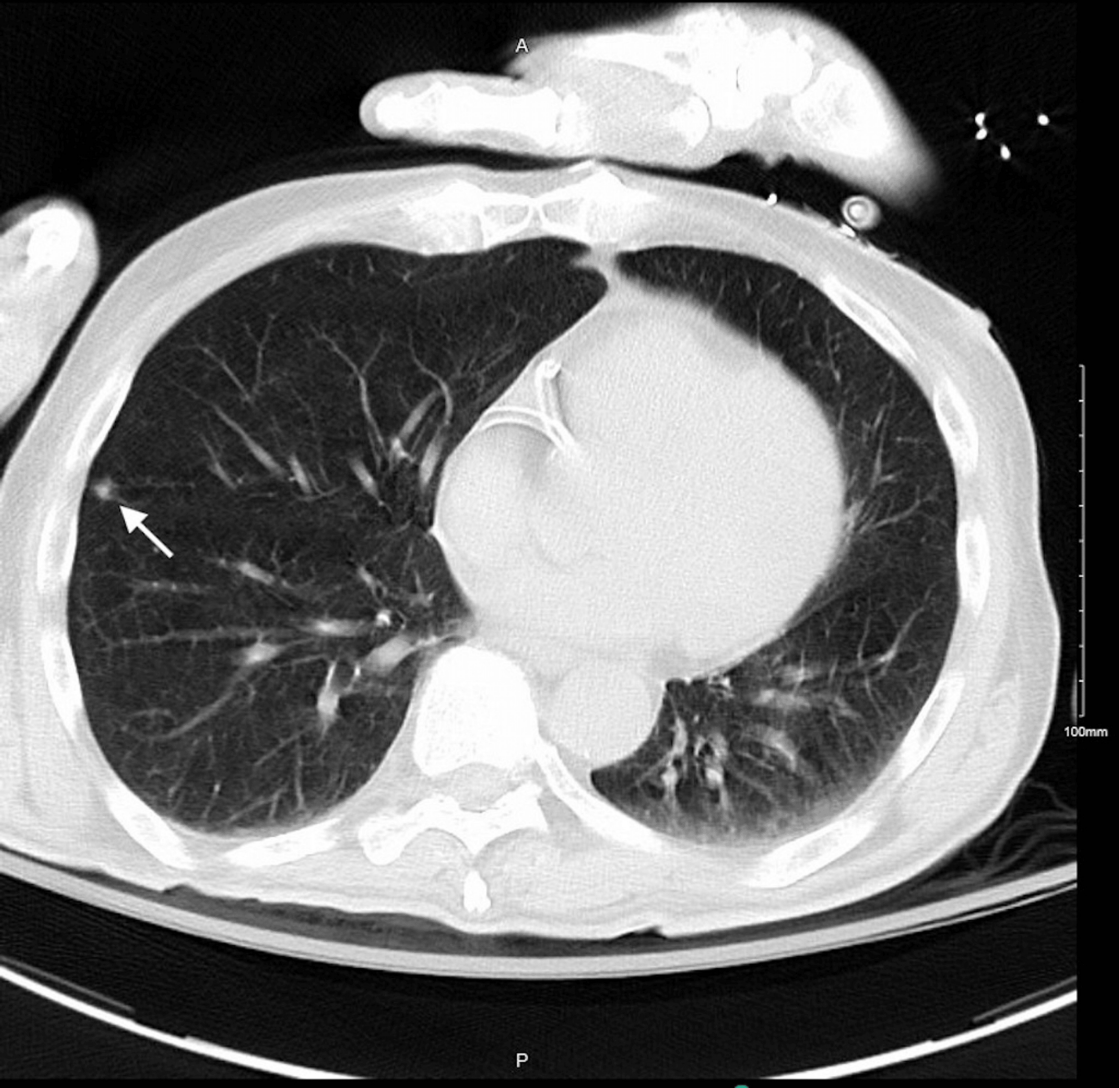
Computed tomography of the chest showing a 6 mm nodule (arrow)

Workup continued as an outpatient, which revealed an elevated 1,25-dihydroxyvitamin D of 84.6 pg/mL (normal range: 18-78 pg/mL). Computed tomography of the abdomen, pelvis, and chest was ordered; however, he returned to the hospital with malaise and worsening hypercalcemia. He was again hemodynamically stable with an unremarkable physical examination, but laboratory findings showed an elevated calcium of 13 mg/dL, creatinine of 2.9 mg/dL, low intact PTH below 6 pg/mL, and elevated 1,25-dihydroxyvitamin D of 96 pg/mL. Urinalysis showed 2+ protein. Computed tomography of the chest revealed a small sub-6 mm nodule in the right middle lobe (Figure 1), but no mediastinal or hilar lymphadenopathy, which is not characteristic of sarcoidosis. Computed tomography of the abdomen and pelvis showed stable postoperative changes from right nephrectomy, nonspecific splenomegaly, and prostatomegaly. No lymphadenopathy was noted. A whole-body bone scan was completed, given concern for malignancy, but was negative. Treatment with aggressive intravenous fluid resuscitation and bisphosphonates led to the resolution of the hypercalcemia, and he was discharged with outpatient follow-up.

After discharge, he underwent frequent outpatient physician visits to determine a diagnosis of his hypercalcemia. A whole-body positron emission tomography/computed tomography scan was performed, but it revealed no abnormal metabolic uptake or lymphadenopathy. The patient was again hospitalized about a month after the last admission for a recurrence of hypercalcemia. During this admission, he was noted to have acute encephalopathy of unclear etiology. Hence, a repeat laboratory workup was undertaken, which again showed an elevated 1,25-dihydroxyvitamin D level of 146 pg/mL, suppressed PTH of 8 pg/mL, and a borderline elevated parathyroid hormone-related protein (PTHrP) of 5 pMol/L (normal: <2.5 pMol/L). Prostate-specific antigen was within the normal range of 3 ng/mL (normal range: 0-4 ng/mL), and cancer antigen 19-9 (CA 19-9) was elevated at 53 U/mL (normal range: 0-37 U/mL). Angiotensin-converting enzyme (ACE) level was elevated at 118 U/L (normal range: 8-65 U/L), which raised concern for sarcoidosis, although classic sarcoidosis findings were absent. Given the patient's history of a solitary kidney, which posed a high complication risk for a kidney biopsy, a bone marrow biopsy was done first to rule out malignancy and possibly sarcoidosis. Bone marrow biopsy revealed trilineage hyperplasia. The myeloid lineage showed progressive maturation with no increase in blasts. There was no increase in lymphocytes or plasma cells, and no evidence of malignancy or sarcoidosis.

**Figure 2:**
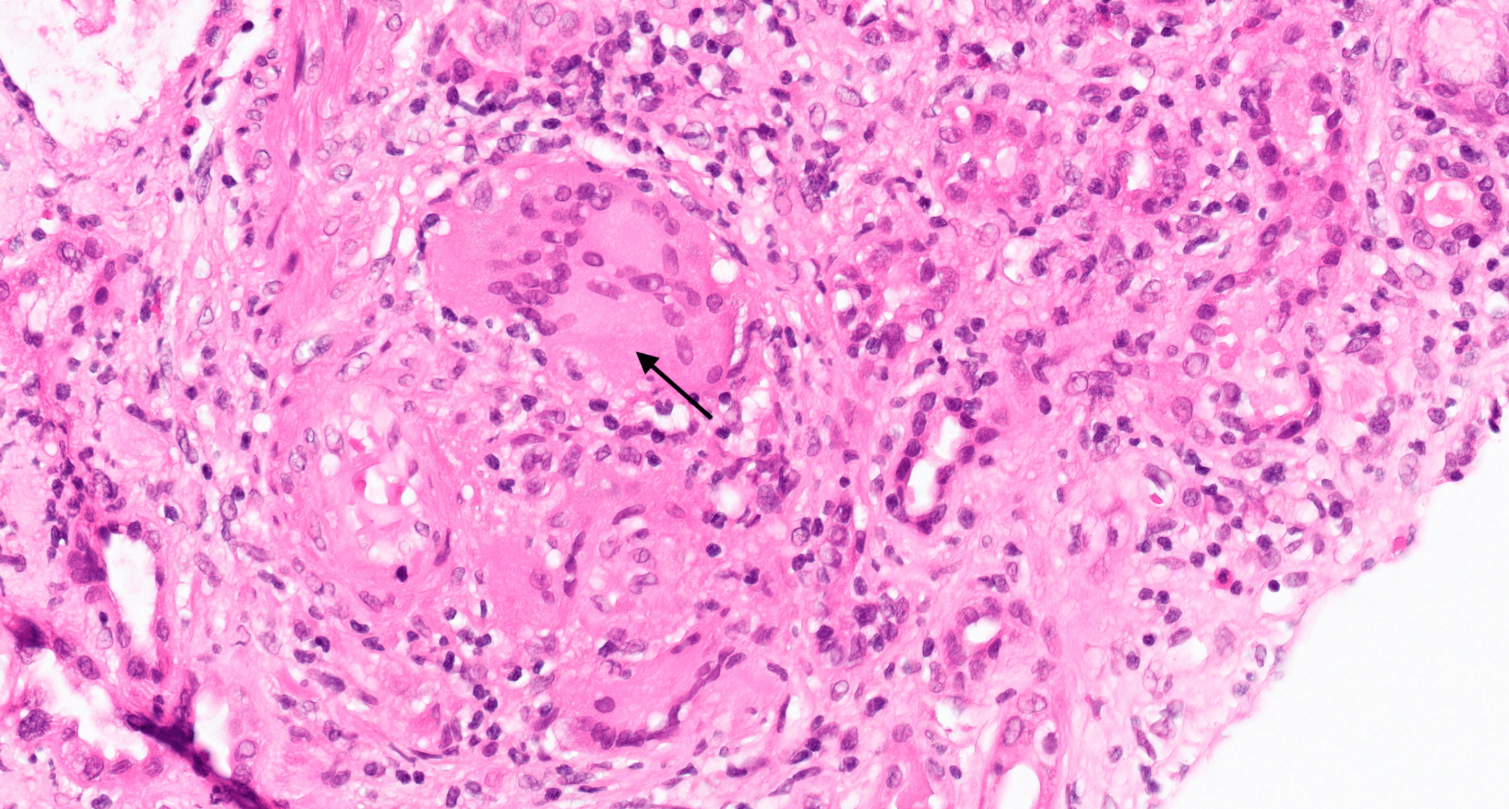
Interstitial granuloma with multinucleated giant cells (arrow) (H&E, ×600) H&E: hematoxylin and eosin

Throughout this time, the patient's kidney function continued to decline, and unfortunately, hemodialysis had to be initiated, which sparked an urgent need for a kidney biopsy to determine if sarcoidosis was at play. The high-risk biopsy revealed tubulointerstitial nephritis, granulomatous inflammation with giant cells (Figure 2), scattered calcium phosphate deposits, and acute tubular necrosis, with possible moderate underlying interstitial fibrosis and tubular atrophy.

These findings were consistent with renal-limited sarcoidosis, and the patient was started on high-dose steroids, after which his overall status began to improve, and he was successfully discharged home. Unfortunately, his renal function did not improve sufficiently enough in order to stop renal replacement therapy.

## Discussion

Sarcoidosis is an uncommon disease, but in most cases, it presents with recognizable systemic features that help facilitate diagnosis. In contrast, this patient lacked many of the typical manifestations, particularly pulmonary involvement, making the diagnostic process significantly more challenging.

Some of the less common findings in sarcoidosis include hypercalcemia, which is associated with a worse prognosis, and splenomegaly, observed in fewer than 10% of cases [6-8]. In such presentations, activated granulomatous macrophages increase conversion of vitamin D to its active form (1,25-dihydroxyvitamin D), thereby enhancing gastrointestinal calcium absorption and suppressing parathyroid hormone (PTH) secretion [7,8]. Another relatively uncommon feature is an elevated angiotensin-converting enzyme (ACE) level, present in approximately 40%-90% of cases [8].

In this case, the patient's combination of atypical findings (hypercalcemia, elevated vitamin D, splenomegaly, and high ACE level) in the absence of classic systemic or pulmonary symptoms ultimately warranted a kidney biopsy. Histopathological confirmation of renal-limited sarcoidosis led to the initiation of high-dose corticosteroids, the first-line treatment. Unfortunately, due to delayed diagnosis and ongoing renal decline, the patient progressed to end-stage renal disease requiring dialysis, underscoring the potential severity of this rare sarcoidosis variant.

## Conclusions

In conclusion, this case illustrates the diagnostic complexity of sarcoidosis, particularly its rare renal-limited form. The absence of typical systemic features significantly delayed diagnosis and contributed to progression to end-stage renal disease. This case underscores the importance of maintaining a high index of suspicion for sarcoidosis in patients presenting with unexplained hypercalcemia, elevated 1,25-dihydroxyvitamin D levels, and renal dysfunction, even in the absence of classic clinical findings.
